# Modulatory Effects of Chalcone Thio-Derivatives on NF-κB and STAT3 Signaling Pathways in Hepatocellular Carcinoma Cells: A Study on Selected Active Compounds

**DOI:** 10.3390/ijms251910739

**Published:** 2024-10-05

**Authors:** Katarzyna Papierska, Eliza Judasz, Wiktoria Tonińska, Maciej Kubicki, Violetta Krajka-Kuźniak

**Affiliations:** 1 Department of Pharmaceutical Biochemistry, Poznan University of Medical Sciences, Rokietnicka 3, 60-806 Poznań, Poland; judasz.eliza@gmail.com (E.J.); wiktoria.toninska99@gmail.com (W.T.); vkrajka@ump.edu.pl (V.K.-K.); 2 Faculty of Chemistry, Adam Mickiewicz University in Poznań, Uniwersytetu Poznańskiego 8, 61-712 Poznań, Poland; maciej.kubicki@amu.edu.pl

**Keywords:** anakoinosis, apoptosis, p53, TNF-α, STAT3, NF-κB, thio-chalcone derivatives, hepatocellular carcinoma cells

## Abstract

Our previous studies demonstrated the modulatory effects of new synthetic thio-chalcone derivatives in dishes on the Nrf2, NF-κB, and STAT3 signaling pathways in colon cancer cells. This study aimed to evaluate the effect of four selected active chalcone thio-derivatives on the NF-κB and STAT3 signaling pathways involved in inflammatory processes and cell proliferation in human liver cancer cells. Cell survival was assessed for cancer (HepG2) and normal (THLE-2) cell lines. Activation of NF-κB and STAT3 signaling pathways and the expression of proteins controlled by these pathways were estimated by Western blot, and qRT-PCR assessed the expression of NF-κB and STAT3 target genes. We also evaluated the impact on the selected kinases responsible for the phosphorylation of the studied transcription factors by MagneticBead-Based Multiplex Immunoassay. Among the thio-derivatives tested, especially derivatives **1** and **5**, there was an impact on cell viability, cell cycle, apoptosis, and activation of NF-κB and STAT3 pathways in hepatocellular carcinoma (HCC), which confirms the possibilities of using them in combinatorial molecular targeted therapy of HCC. The tested synthetic thio-chalcones exhibit anticancer activity by initiating proapoptotic processes in HCC while showing low toxicity to non-cancerous cells. These findings confirm the possibility of using chalcone thio-derivatives in molecularly targeted combination therapy for HCC.

## 1. Introduction

Liver cancer is a highly heterogeneous cancer that remains one of the most difficult oncology challenges. Inflammatory mediators, whose activators or targets are transcriptional factors such as Signal Transducer and Activator of Transcription 3 (STAT3), and Nuclear Factor kappa B (NF-κB) play a vital role in the development of carcinogenesis hepatocellular carcinoma (HCC) [1]. In normal cells, the NF-κB is a set of dimeric subunits in the cytoplasm bound to inhibitory IkB proteins. Chronic inflammation is associated with the high incidence of HCC, characterized by increased NF-κB activity and overexpression of genes encoding proteins involved in critical cellular processes, such as proliferation, apoptosis, differentiation, and cell adhesion. The binding of NF-κB family members to DNA initiates the transcriptional activity of the cellular inhibitor of apoptosis genes, the antiapoptotic protein B-cell lymphoma extra large (Bcl-xL), and proapoptotic protein bcl-2-like protein 4 (Bax). The influence of NF-κB on the development of hepatocarcinogenesis depends primarily on the type of cell and the degree of activation or inhibition of NF-κB. The most potent activators of NF-κB are liposaccharides, viral or bacterial DNA and RNA, and inflammatory cytokines such as Tumor Necrosis Factor (TNF) or Interleukin-1 (IL-1). They lead to the transcription of genes responsible for regulating inflammation, immune response, and cell survival [2,3].

We also observe abnormal overexpression and activation of the cytoplasmic transcription factor STAT3 during tumor progression. By binding to the appropriate receptor, cytokines and growth factors such as IL-1 activate Janus kinases responsible for STAT3 phosphorylation. Clinical studies have shown that STAT3 overexpression, occurring in 60% of HCC cases in vitro, is associated with worse disease prognosis, including larger tumor size, increased tissue vascularization, and higher migration potential [1]. According to the present research, it is suggested that the effect of some chalcone derivatives is a point of capture for the NF-κB and STAT3 pathways modulators [4]. Chalcones are polyphenols widely distributed in fruits, vegetables, tea, and spices, such as hops and licorice. They are also precursors of secondary metabolites for the synthesis of flavonoids and isoflavonoids. Chemically, they are open-chain flavonoids in which two aromatic rings are connected by a three-carbon α,β-unsaturated carbonyl system (1,3-diphenyl-2-propen-1-ones) [5,6]. Natural chalcones exhibit a range of biological activities, including anticancer, antiangiogenic, anti-inflammatory, antioxidant, immunomodulatory, antibacterial, antimalarial, and analgesic activities [5,6,7,8]. They can induce apoptosis and cell cycle arrest or inhibit tumor promotion and metastasis in cancer cells [9]. These activities are mainly attributed to the presence of the α,β-unsaturated carbonyl system in chalcones, which can act as a Michael acceptor (leading to the formation of a new carbon–carbon bond).

This moiety readily forms covalent bonds with nucleophiles, such as thiol cysteine residues present in peptides or cellular proteins, to obtain a Michael adduct, which may play an essential role in the molecule’s biological activity [10]. In our previous studies, we used the synthetic derivatives of chalcone with structural modifications involving the replacement of the divalent oxygen atom with sulfur to improve their anticancer activity. Studies have proven the anticancer activity of synthetic thio-chalcone derivatives in colorectal cancer cells of the DLD-1 and HCT116 lines, consisting of modulating the NF-κB, STAT3, and Nrf2 signaling pathways. Eight thio-chalcones underwent extensive biological analysis during in vitro tests, thereby identifying the four most active compounds against colorectal cancer cells. Among these, derivatives **4** and **5** notably reduced NF-κB activation and COX-2 target gene expression. For STAT3, inhibition of this pathway’s activation was observed only with derivative **4**. Based on these data, the most active chalcone thio-derivatives (**1**, **2**, **4**, and **5**) were selected for study to assess their potential anticancer properties against HepG2 cells [11]. Thio-chalcones exhibit affordable bioavailability and limited toxicity towards healthy cells, making them a promising agent also against HCC cells.

This article presents the research results using the four selected most active thio-derivatives of chalcones in hepatocellular carcinoma cells (HepG2) (Table 1).

Our analysis tries to explain and confirm the biological properties of selected thio-chalcones as modulators of the expression of transcription factors involved in the process of carcinogenesis of HCC cells. Such combinatorial molecular targeting fits into the trend called anakoinosis, which means “communication”. According to anakoinosis, induced signaling in cancer cells aims to establish characteristic communication between cancer tissues or between cancer tissue and the host organism. It occurs due to the modulation of the expression of at least two factors simultaneously [12].

## 2. Results

### 2.1. The Influence of the Tested Thio-Chalcones on the Viability Cells

**Figure 1:**
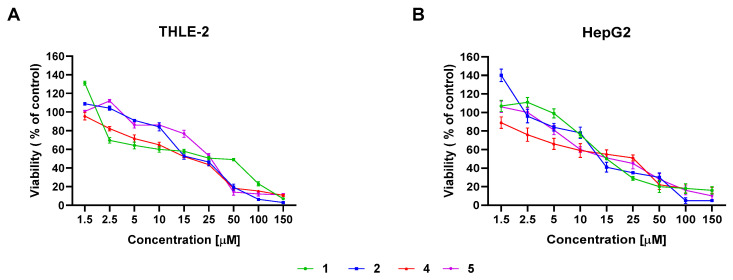
The cytotoxicity evaluation of synthetic thio-chalcone derivatives (**1**, **2**, **4**, and **5**) on: (**A**) THLE-2 cell line and (**B**) HepG2 cell line. Control cells were treated with the vehicle. The values are shown as the mean ± SEM calculated from three independent experiments. The colors and the numbers, respectively, mean the tested synthetic chalcone thio-derivatives: **1**—3-(4-methoxy-3-methylthiophenyl)-1-(3,4,5-trimethoxyphenyl)-prop-2-en-1-one. **2**—3-(3-methoxy-4-methylthiophenyl)-1-(3-bromo-4,5-dimethoxyphenyl)-prop-2-en-1-one. **4**—3-(4-methylthiophene)-1-(3-bromo-4,5-dimethoxyphenyl)-prop-2-en-1-one. **5**—3-(3-methoxy-4-methylthiophenyl)-1-(3-bromo-5-methoxy-4-methylthiophene)-prop-2-en-1-one.

The viability of cancerous hepatocytes (HepG2) was assessed within the range of 1 µM to 150 µM. As Figure 1 illustrates, the viability of HepG2 cells within the range of up to 50 µM was similar for the tested derivatives.

Moreover, normal hepatocytes (THLE-2) were also used comparatively to determine the impact of the tested derivatives. Derivatives **1** and **5** exhibited higher cytotoxicity towards THLE-2 cells than against the HepG2 cells. In turn, HepG2 cells were more susceptible than THLE-2 to derivatives **2** and **4** cytotoxic activity. Based on the MTT results and the IC50 values (Table 2), further investigations were selected for the concentrations 5 µM (which are marked on the figures as **1**/5; **2**/5; **4**/5; **5**/5) and 15 µM (which are marked on the figures as **1**/15; **2**/15; **4**/15; **5**/15) of thio-derivatives of chalcones to assess the cell cycle distribution, apoptosis profile, activity, and expression of the transcription factors NF-κB and STAT3 pathways in the HepG2 cell line.

### 2.2. The Changes in the Distribution of Cell Cycle Phases and the Apoptotic Cell Death after the Treatment with the Tested Thio-Chalcones

**Figure 2:**
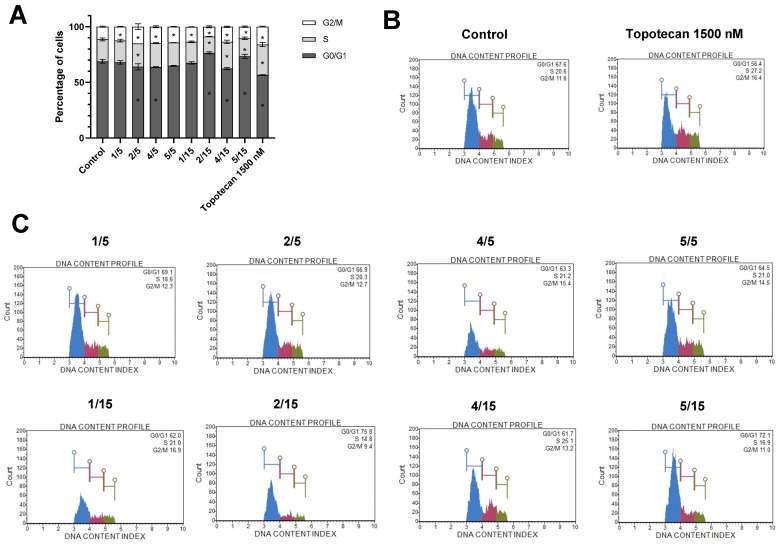
Distribution of cell cycle phases in HepG2 cell line after 24 h incubation with synthetic thio-chalcone derivatives (**1**/5, **2**/5, **4**/5, **5**/5—thio-chalcone/at a concentration of 5 μM; **1**/15, **2**/15, **4**/15, **5**/15—thio-chalcone/at a concentration of 15 μM). (**A**) Graphs of the mean ± SEM of the percentage of cells in G1/G0, S, and G2/M phases were calculated from two independent experiments. (*) indicates statistically significant differences compared to the control group for a given phase (*p* < 0.05). (**B**) Histograms of the negative (DMSO) and positive (Topotecan 1500 nM) control analysis. (**C**) Histograms of representative samples for individual compounds.

To investigate the effect of the thio-derivatives chalcones on cell cycle distribution in HepG2 cells, assessment after 24 h of treatment with the analyzed compounds was conducted (Figure 2).

Similar to the positive control (Topotecan), compound **2** at a concentration of 5 µM and 15 µM and derivatives **4** and **5** at a concentration of 15 µM caused cell redistribution in all analyzed cell cycle phases, leading to cell cycle arrest in the G2/M phase.

**Figure 3:**
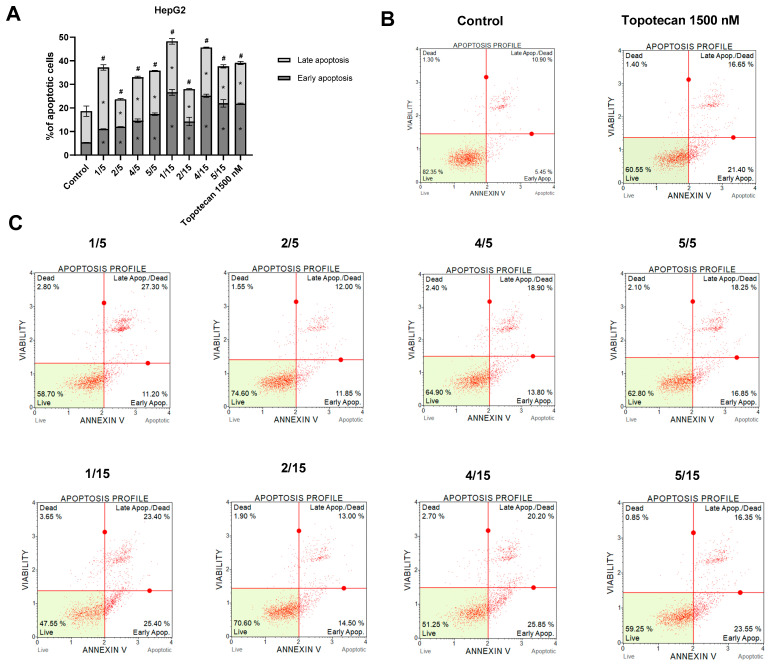
Apoptosis profile of HepG2 cell line treated with the synthetic thio-chalcone derivatives (**1**/5, **2**/5, **4**/5, **5**/5—thio-chalcone/at a concentration of 5 μM; **1**/15, **2**/15, **4**/15, **5**/15—thio-chalcone/at a concentration of 15 μM). (**A**) Graphs of the mean ± SEM of the percentage of apoptotic cells (in the early, late, and complete phases) after 24 h of incubation with the test compounds. Statistically significant differences compared to the control group of early apoptosis * (*p* < 0.05) and the control group of late apoptosis # (*p* < 0.05). (**B**) Histograms of the negative control (DMSO) and positive (Topotecan 1500 nM) analysis. (**C**) Histograms of representative samples for individual compounds. The four square markers of each graph reflect different cellular states: the top left square contains dead cells (necrosis), the top right contains late apoptosis/dead cells (cells that are positive for both Annexin V and the cell death marker 7-AAD), the left lower corner contains living cells, and the lower right corner contains early apoptosis cells (cells that are positive only for annexin V).

Flow cytometry analysis showed that treating HepG2 cells with synthetic chalcone thio-derivatives induces apoptotic cell death (Figure 3). The most significant effects were obtained as a result of the use of compounds **1** and **4** at a concentration of 15 µM, which increased the percentage of apoptotic cells to an even greater extent than the positive control (Topotecan). Moreover, the remaining compounds showed similar effects, increasing the percentage of apoptotic cells. In summary, early, late, and total apoptosis was increased after treatment with the analyzed compounds, compared to control cells treated with DMSO.

**Figure 4:**
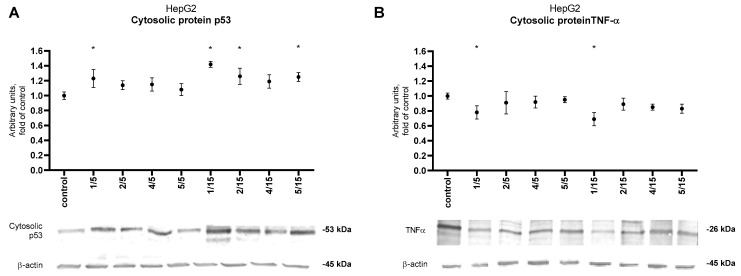
Effect of the tested synthetic thio-chalcone derivatives (**1**, **2**, **4**, and **5**) in the HepG2 cell line on the protein level of: (**A**) p53 and (**B**) TNF-α.Protein levels were expressed as relative changes in the protein level with respect to the control. Representative Western blots of cytosolic fraction treated anti-p53 and anti-TNF-α antibodies are presented under the graphs. The order of the bands in the immunoblot image corresponds to the order of the bars in the diagram. Anti-β-actin antibodies were used to normalize the results. The results presented are the mean ± SEM from two separate experiments. Statistically significant differences compared to the control group * (*p* < 0.05). See also Supplementary Figure S1.

The p53 protein level analysis (Figure 4A) showed increased expression after 24-h incubation for all tested samples. However, only thio-derivative **1** significantly increased the level of this protein at both concentrations used. Moreover, a statistically significant increase of p53 was observed for all derivatives at a concentration of 15 µM except derivative **4**. Furthermore, the results obtained for thio-derivative **1** about p53 correlated with a reduced level of TNF-α protein (Figure 4B) also at 5 µM and 15 µM.

### 2.3. Bead-Based Multiplex Immunoassay as a Screening Test to Examine the Possibility of Modulation of Protein Regulating Several Signaling Pathways by the Tested Thio-Chalcones

**Figure 5:**
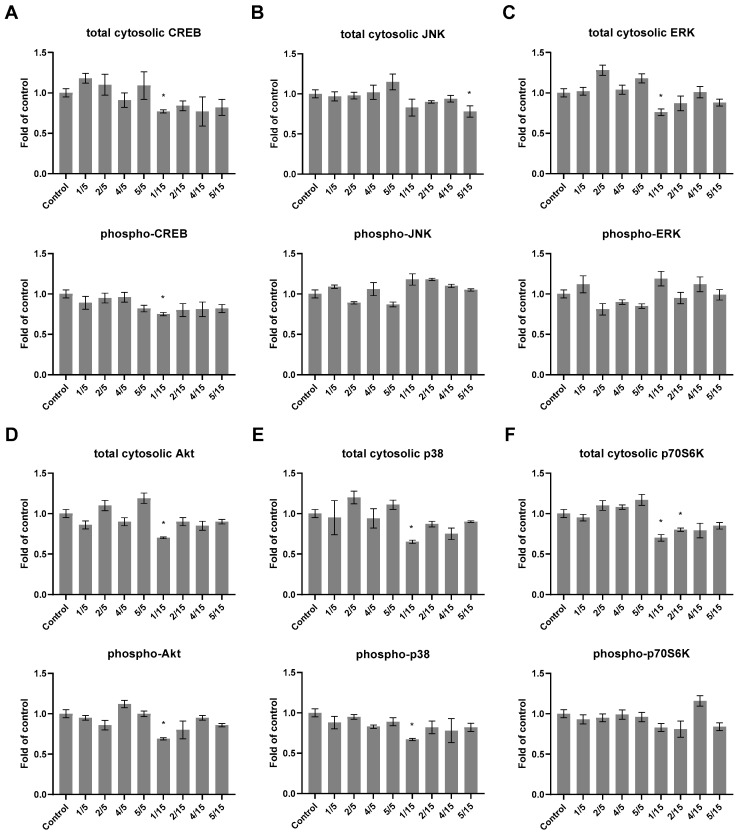
Effect of synthetic thio-chalcone derivatives (**1**, **2**, **4**, and **5**) on the regulation of protein controlling several signaling pathways measured by bead-based multiplex immunoassay in HepG2 cell line. The relative changes in the protein level of: (**A**) CREB and phospho-CREB, (**B**) JNK and phospho-JNK, (**C**) ERK and phospho-ERK, (**D**) Akt and phospho-Akt, (**E**) p38 and phospho-p38 and (**F**) p70S6K and phospho-p70S6K were measured. Results are prepared based on the cytosolic fraction of proteins and are shown in comparison to vehicle control. The values are shown as the mean ± SEM calculated from two independent experiments (a fold of control). Statistically significant differences compared to the control group * (*p* < 0.05).

It is well known that changes in one signaling pathway can significantly disrupt cellular homeostasis. To investigate the possible interactions of thio-derivatives of chalcones with signaling pathways related to cell proliferation and apoptosis, a multiplex bead-based immunoassay in HepG2 cells was performed (Figure 5).

The cytosolic levels of the cAMP response element binding protein (CREB) and Jun N-terminal kinase (JNK), extracellular signal-regulated kinases (ERK), mitogen-activated protein kinases (p38), serine/threonine protein kinase (Akt), ribosomal protein S6 kinase β-1 kinase (p70S6K), and their phosphorylated forms were assessed. A significant reduction in the levels of ERK, p38, Akt, and p70S6K proteins resulted from the treatment of HepG2 cells with derivative **1** at a concentration of 15 µM, which was also reflected in the phosphorylated form of p38 and Akt kinases. In turn, the CREB and phospho-CREB protein levels were reduced (Figure 5A) under the influence of derivative **1**.

### 2.4. The Activation of the STAT3 Signaling Pathway after the Treatment with the Tested Thio-Chalcones

**Figure 6:**
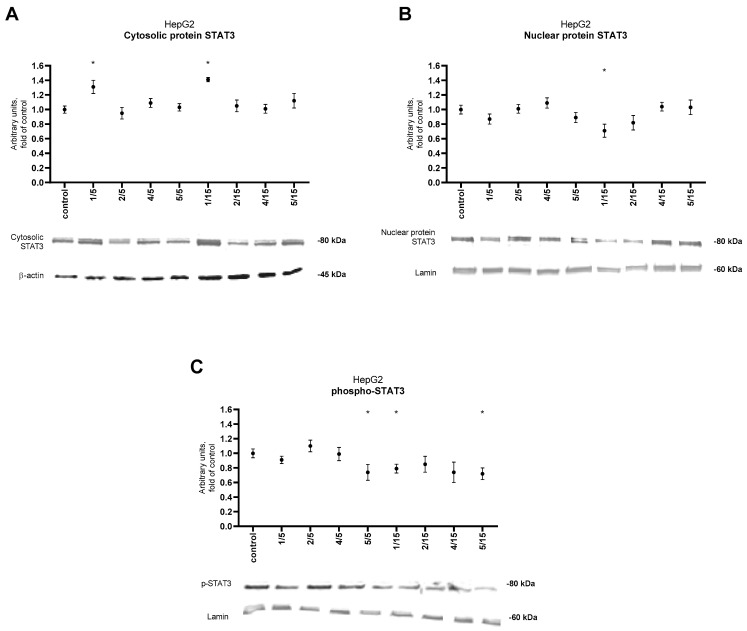
Effect of the tested synthetic thio-chalcone derivatives (**1**, **2**, **4**, and **5**) on the protein level of: (**A**) STAT3 in cytosolic fraction from the HepG2 cell line, (**B**) STAT3 in nuclear fraction from the HepG2 cell line and (**C**) phospho-STAT3 in nuclear fraction from the HepG2 cell line. Protein levels were expressed as relative changes in the protein level with respect to the control. Representative Western blots of cytosolic fraction-treated anti-STAT3 and nuclear fraction-treated anti-STAT3 and anti-phospho-STAT3 antibodies are presented under the graphs. The order of the bands in the immunoblot image corresponds to the order of the bars in the diagram. Anti-β-actin and anti-lamin antibodies were used to normalize the results. See also Supplementary Figure S2. The results presented are the mean ± SEM from two separate experiments. Statistically significant differences compared to the control group * (*p* < 0.05).

Phosphorylation of the tyrosine residue of STAT3 monomers leads to their dimerization and translocation to the cell nucleus. The analysis of the STAT3 protein level in the cytosolic and nuclear fractions, aimed at assessing the impact of the tested compounds on STAT3 translocation, is shown in Figure 6A,B.

Thio-derivative **1** at a concentration of 5 μM and 15 μM caused a significant increase in the level of STAT3 protein in the cytosolic fraction compared to the control cells. These results also coincided with reduced levels of the STAT3 factor in the nuclear fraction only for thio-derivatives **1**. The phospho-STAT3 protein level was affected by derivative **5** at both concentrations used and by derivative **1** at 15 μM, causing its significant reduction (Figure 6C). This may indicate the ability of the derivatives mentioned above to inhibit the phosphorylation of STAT3, i.e., an active form of STAT3.

**Figure 7:**
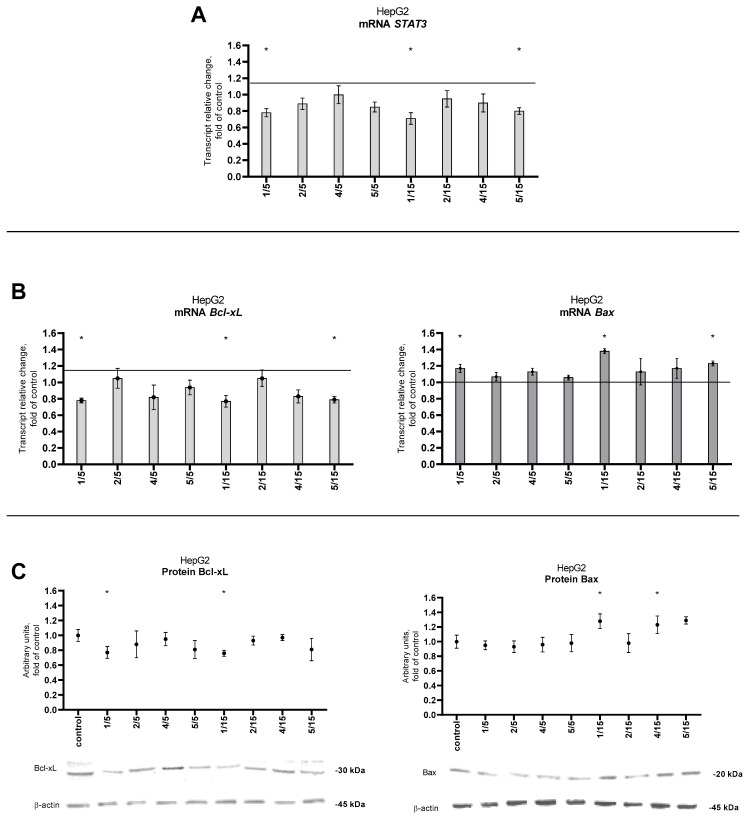
Effect of the tested synthetic thio-chalcone derivatives (**1**, **2**, **4**, and **5**) on the expression of STAT3 and selected target genes in the HepG2 cell line: (**A**) STAT3 transcript level and (**B**) Bcl-xL and Bax transcript levels. The transcript level was calculated as the mRNA level compared to control cells, for which expression was considered. The results presented are the mean ± SEM from two separate experiments. Statistically significant differences compared to the control group * (*p* < 0.05). (**C**) Bcl-xL and Bax protein levels. Protein levels were expressed as relative changes in the protein level with respect to the control. Representative Western blots of cytosolic fraction-treated anti-Bcl-xL and anti- Bax antibodies are presented under the graphs. The order of the bands in the immunoblot image corresponds to the order of the bars in the diagram. Anti-β-actin antibodies were used to normalize the results. See also Supplementary Figure S3. The results presented are the mean ± SEM from two separate experiments. Statistically significant differences compared to the control group * (*p* < 0.05).

Assessment of STAT3 gene expression showed that, similarly to the protein, derivative **1** significantly reduced the STAT3 transcript level at both tested concentrations (Figure 7A).

Also, thio-chalcone **5** at 15 μM showed a reduction effect on STAT3 expression. The inhibitory effect of these two derivatives was also demonstrated by analyzing the expression of selected STAT3 target genes: antiapoptotic protein—Bcl-xL and proapoptotic protein—Bax (Figure 7B,C).

### 2.5. The Activation of the NF-κB Signaling Pathway after the Treatment with the Tested Thio-Chalcones

The NF-κB transcription factor is the most common dimeric complex protein from p50/p65 subunits. NF-κB activation occurs as a result of phosphorylation of the IκB inhibitor protein via two kinase subunits, IKKα and IKKβ. This leads to the degradation of IκB and, consequently, the translocation of active NF-κB dimers to the cell nucleus.

**Figure 8:**
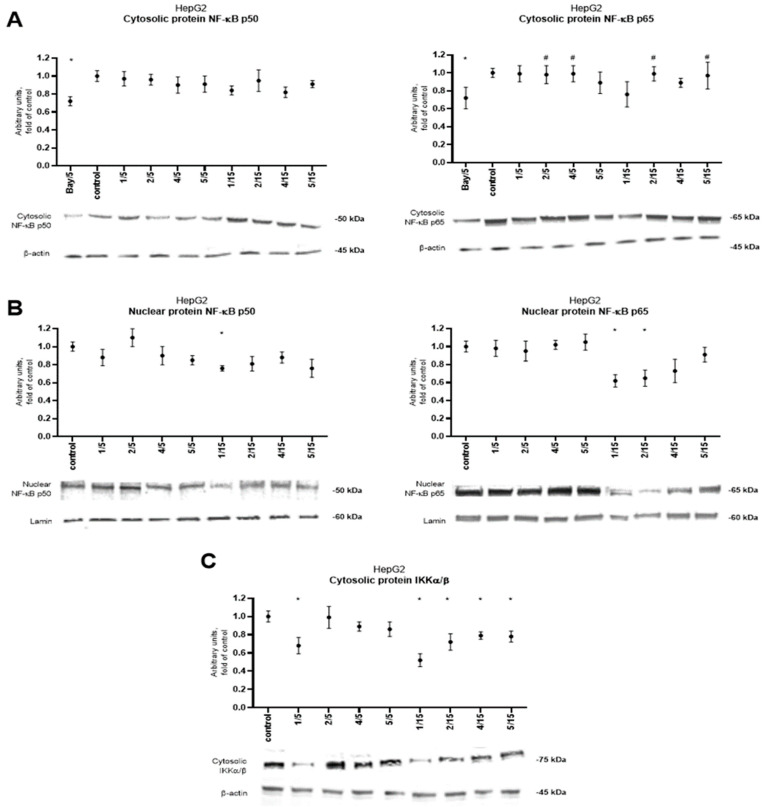
Effect of the tested synthetic thio-chalcone derivatives (**1**, **2**, **4**, and **5**) on the protein level of: (**A**) NF-κB p50 and p65 subunits in cytosolic fraction from HepG2 cell line, (**B**) NF-κB p50 and p65 subunits in nuclear fraction from HepG2 cell line and (**C**) IKKα/β in cytosolic fraction from HepG2 cell line. Protein levels were expressed as relative changes in the protein level with respect to the control. Representative Western blots of cytosolic and nuclear fraction-treated anti-NF-κB p50 and anti-NF-κB p65 and cytosolic fraction-treated anti-IKKα/β antibodies are presented under the graphs. The order of the bands in the immunoblot image corresponds to the bars in the diagram. Anti-β-actin and anti-lamin antibodies were used to normalize the results. See also Supplementary Figure S4. The results presented are the mean ± SEM from two separate experiments. Statistically significant differences compared to the control group * (*p* < 0.05) and compared to the BAY 11-7082 # (*p* < 0.05).

After analyzing changes in the level of NF-κB in HepG2 cells treated with thio-chalcones, the effect of thio-derivative **1** at a concentration of 15 µM in the nuclear fraction reduced the level of both p65 and p50 subunits (Figure 8B) and had the most potent effect on the inhibitor protein level among all the tested derivatives (Figure 8C). In turn, derivative **2** at a concentration of 15 µM on the p65 subunit could be observed. We also noticed a decrease in the IKKα/β protein level after using thio-chalcone **1** at 5 µM, and all tested thio-chalcones at higher concentrations (Figure 8C).

The compound BAY 11-7082 was also used in the study to compare the effects of the tested thio-derivatives of chalcones with the effects of a known NF-κB inhibitor. In HepG2 cells, a decrease in the level of the transcription factor protein was observed to a greater extent under the influence of BAY 11-7082 than due to the action of the tested thio-chalcones (Figure 8A). However, statistically significant results relative to BAY 11-7082 as a positive control were only observed for the cytosolic fraction of p65 in the case of derivatives **2** and **4** at a concentration of 5 µM and **2** and **5** at a concentration of 15 µM.

**Figure 9:**
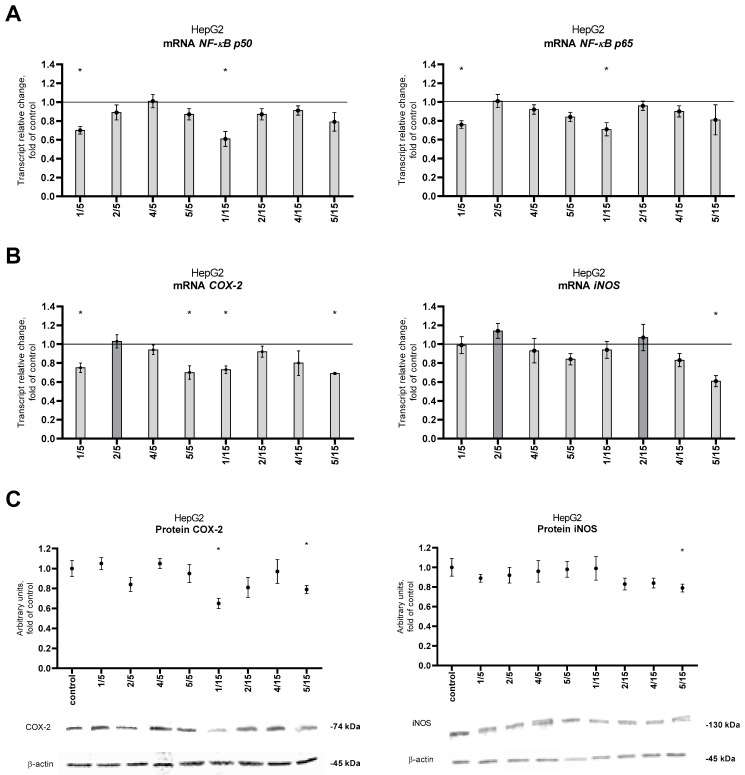
Effect of the tested synthetic thio-chalcone derivatives (**1**, **2**, **4**, and **5**) on the expression of NF-κB p50 and p65 subunits and selected target genes in the HepG2 cell line. (**A**) NF-κB p50 and p65 subunit transcript levels, and (**B**) COX-2 and iNOS transcript levels were measured. The transcript level was calculated as the mRNA level compared to control cells, for which expression was considered 1. The results presented are the mean ± SEM from two separate experiments. Statistically significant differences compared to the control group * (*p* < 0.05). (**C**) COX-2 and iNOS protein levels. Protein levels were expressed as relative changes in the protein level with respect to the control. Representative Western blots of cytosolic fraction-treated anti-COX-2 and anti-iNOS antibodies are presented in the graphs. The order of the bands in the immunoblot image corresponds to the order of the bars in the diagram. Anti-β-actin antibodies were used to normalize the results. See also Supplementary Figure S5. The results presented are the mean ± SEM from two separate experiments. Statistically significant differences compared to the control group * (*p* < 0.05).

NF-κB induction results from the release of active subunits from the cytoplasmic complex with IκB and induction of the expression of genes encoding these subunits. The expression level of NF-κB p50 and p65 at the mRNA level in HepG2 cells was significantly reduced under derivative **1** at a concentration of 5 μM and 15 µM (Figure 9A). The effect of chalcones on the expression of NF-κB target genes COX-2, and inducible nitric oxide synthase (iNOS) was also assessed. Derivative **1** and derivative **5** at 5 μM and 15 μM caused a significant reduction in the level of COX-2 transcript, which was confirmed at the protein level only after using a higher concentration (Figure 9B,C). Additionally, thio-chalcone **5** at 15 μM also reduced iNOS expression at both the transcript and protein levels (Figure 9B,C).

## 3. Discussion

Liver cancer has one of the worst prognoses, which confirms the need for continuous research to search for new therapies. Therefore, our study focused on evaluating synthetic thio-derivatives in the context of modifications to signaling pathways critical for hepatic cell survival and carcinogenesis. Natural chalcones have several biological activities, including anticancer, antiangiogenic, anti-inflammatory, antioxidant, immunomodulatory, antibacterial, antimalarial, and analgesic. Chalcones can induce apoptosis, cell cycle arrest, or inhibit tumor promotion and metastasis in cancer cells, like mammary glands, hematopoietic systems, or germ cells. In vivo studies have also demonstrated their effectiveness as chemopreventive agents. These properties make chalcones a promising area of research for developing new cancer treatments and preventive strategies [5,11].

However, the major challenge is developing effective anticancer drugs with noor low toxicity and minimum acute and long-term side effects on normal tissues. One way to address these challenges may be targeting cancer hallmarks, e.g., with bioactive chalcones.

Based on data on colorectal cancer cells (DLD-1 and HCT116 lines), in this work, we focused on the most biological activity from synthetic chalcone thio-derivatives on hepatoma cells (HepG2 line). Hepatocellular carcinoma and colorectal cancer are tumors whose carcinogenic process is accompanied by a progressive inflammatory process, which additionally supports the aggressive progression of the disease. This is primarily related to the epidemiology of the disease, i.e., the consequences of Helicobacter pylori, viral hepatitis B/C, and inflammatory bowel disease (IBD) [13]. Logan et al. demonstrated that xanthohumol has antitumor activity with delivery-dependent cytotoxicity in in vitro studies on the HepG2, Huh7, HCT116, and HT29 cell lines. The IC50 in colon cancer cell lines was higher than in the pathological liver cell lines, confirming that HCT116 and HT29 cells are more active due to the action of the natural chalcone [14]. HepG2 cells are an in vitro alternative to primary human hepatocytes. They are characterized by an unlimited lifespan, stable phenotype, and high availability. However, their main limitation is the lower expression of some metabolic activities compared to hepatocytes. HepG2 is most commonly used in drug metabolism and hepatotoxicity studies. HepG2 cells are non-neoplastic cells with a high proliferation rate and epithelial-like morphology that perform diverse liver functions [15].

The current results showed that derivatives **1** and **5** significantly increased toxicity against cancer HepG2 than immortalized non-tumor THLE-2 cells. Our team’s research showed that xanthohumol had a similar effect, a more significant toxic effect on the HepG2 line than the THLE-2, and chalcone thio-derivative **1** had a more cytotoxic influence on HCT116 and DLD-1 lines than on the normal human HaCaT cells [11,16].

Several studies indicated that HCC characterizes the dysregulation of essential cellular mechanisms, including those related to cell cycle, differentiation, apoptosis, and signaling pathways [17,18]. Cytometric analyses of cell cycle distribution showed for the tested thio-derivatives **2**, **4**, and **5** a redistribution of cells in all analyzed cell cycle phases, similar to the case of Topotecan, i.e., the positive control. Moreover, all thio-chalcones at both concentrations decreased the number of cells in the G2/M phase. The obtained results correlate with the presented effects of thio-chalcones on colorectal cancer cells, where changes in the redistribution of cell cycle phases were also observed for derivatives **4** and **5** at the higher concentrations used (10 µM) [11]. Furthermore, a similar influence on cell cycle distribution was recorded for xanthohumol at a concentration of 20 µM in HepG2 line [19]. These findings suggest that the tested synthetic thio-chalcones can initiate the programmed cell death process in HCC cells, supporting their potential use in cancer therapy where there is a disturbed balance between apoptosis and cell proliferation processes [20,21].

Furthermore, all tested thio-derivatives induced apoptosis, which resulted in changes in the level of proteins associated with this process. Thio-derivatives **4** and **5**, already at a dose of 5 µM in colorectal cancer cells, also induced a higher percentage of late and total apoptosis than BAY 11-7082 [11]. One of the most essential suppressors of cancer transformation related to the apoptosis process is the p53 factor. By accumulating in the structure of the cell nucleus, the p53 protein stops the cell cycle, promotes DNA repair, or puts the cell on the apoptosis track [22,23]. Conversely, TNF-α is a proinflammatory cytokine produced by immune system cells and cancer cells. In HCC cells, TNF-α has been characterized as a pro-tumor factor because its increased expression is associated with the activation of the NF-κB and JNK pathways, promoting carcinogenesis and supporting cancer cell survival and migration [24,25]. Therefore, searching for compounds with a p53 inducer and TNF-α inhibitor potential is a crucial strategy in treating liver cancer [26]. Our results for HepG2 cells confirm the increase in p53 and decreased TNF-α protein level under the influence of derivative **1**, which depended on used doses.

The next step of our research was the bead-based multiplex immunoassay. This method was chosen for its ability to measure multiple signaling proteins simultaneously with high sensitivity. One key advantage of this method is its efficiency in analyzing multiple pathways in a single experiment compared to Western blotting or ELISA, which typically focuses on one protein at a time. Additionally, the bead-based method provides quantitative data, which is critical for measuring subtle changes in protein expression levels, phosphorylation states, and their roles in cellular responses. This ability to capture a broad spectrum of signaling molecules and their modifications offers a comprehensive understanding of how chalcone thio-derivatives modulate multiple cancer-related pathways. This is essential when studying complex signaling pathways like NF-κB and STAT3, which are involved in cancer progression. The assay allows the detection of various phosphorylated and non-phosphorylated forms of kinases, including ERK, p38, and Akt, which are critical for understanding cellular proliferation and apoptosis in hepatocellular carcinoma cells. These kinases transmit signals to the nucleus, causing cellular responses such as inflammatory processes [27]. Multiple growth factors and inflammatory signals induce CREB and then mediate the transcription of genes containing a cAMP-responsive element. Several immune-related genes have this cAMP-responsive element, including interleukins like IL-2, IL-6, IL-10, and TNF-α protein. Furthermore, it has been proposed that phosphorylated CREB directly inhibits NF-κB activation by blocking the binding of the CREB-binding protein to the NF-κB complex, thereby limiting proinflammatory responses [28]. Serine/threonine kinase (Akt), an oncoprotein, is constitutively active in various cancers and has been suggested to phosphorylate IKK directly, consequently promoting NF-κB-dependent oncogenesis [29]. In turn, several studies have shown that blocking the activity of the p38 protein attenuates the transcriptional activity of the proinflammatory transcription factor NF-κB without changing its DNA-binding activity [30]. The interconnectedness in activating these pathways concerning NF-κB makes them important targets for potential anticarcinogenic and antiapoptotic compounds.

The current study demonstrated that thio-derivative **1** significantly modulated transcription factor CREB and some kinases from the mitogen-activated protein kinase family using multiplex bead-based immunoassay analysis. In another study, we indicated that xanthohumol also regulated the level of ERK kinase in HepG2 cells, with dose-dependent effects [19].

Our previous studies using synthetic thio-derivatives of chalcones in colon cancer cells showed that these compounds inhibit the expression of the p-STAT3 protein, and a reduction in the nuclear fraction of STAT3 binding to DNA was observed [11]. In HCC cells, the STAT3 pathway plays a similar role, but its activation may be more complex due to the tumor microenvironment. HCC develops in the course of chronic hepatitis, which is associated with the continuous production of pro-inflammatory cytokines (TNF-α), which cause excessive activation of the STAT3 pathway, which promotes cancer development (positive feedback loop). Moreover, an association between STAT3 activity and poor prognosis in HCC has been observed. Activated STAT3 in HCC cells promotes proliferation by regulating anti-apoptotic genes (Bcl-2, Bax) and supports angiogenesis (VEGF) [31].

Furthermore, the STAT3 transcription factor interacts with NF-κB in various ways, including activation by NF-κB target gene proteins. The p50 and p65 subunits of the NF-κB complex can interact with STAT3 by facilitating the binding of STAT3 promoters to NF-κB. Additionally, STAT3 may influence the post-translational modification of RelA mediated by p300, resulting in NF-κB acetylation and increased accumulation in the cell nucleus. In turn, NF-κB acetylation causes overexpression of NF-κB, promoting inflammation in the tumor microenvironment [32]. STAT3 and NF-κB regulate the transcription of genes encoding cytokines and chemokines, including IL-6 and TNF-α. They can induce the binding of STAT3 to the membrane receptor for TNF-α, inducing its expression. Meanwhile, TNF-α triggers endogenous IL-6 expression, regulated by NF-κB [33,34].

The conducted research showed that thio-derivatives of chalcones could modulate the NF-κB and STAT3 signaling pathways. The most promising results were obtained for derivative **1** at a concentration of 15 µM, which is probably related to the location of the thiol substituent in the 5-position compared to other tested thio-chalcones.

The use of thio-derivative 1 led to a reduction in the translocation of the STAT3 dimer from the cytosol to the cell nucleus—where the active, i.e., phosphorylated form of STAT3 occurs. However, this effect depends on the thio-chalcone concentration. Moreover, it is confirmed by the results of Western Blot and qRT-PCR analysis for this derivative, which also, at the highest concentration used (15 µM), caused a decrease in the level of phospho-STAT3 and a decrease in STAT3 expression at the transcript level of HepG2 cells. This compound also inhibited the nuclear level of both subunits of NF-κB.

To take a closer look at the impact of the tested compounds on the expression of STAT3 and NF-κB pathways, the expression of selected genes associated with these signaling pathways was analyzed: Bcl-xL, Bax, COX-2, and iNOS. Thio-derivative **1** also significantly influenced the expression of the mentioned genes. A decrease in the level of the antiapoptotic protein Bcl-xL was observed in HepG2 cells, which correlated with an increase in the level of the proapoptotic protein Bax. Moreover, a decrease in COX-2 gene expression was noted, which was confirmed by the results of Western Blot and qRT-PCR analyses. Moreover, tested derivatives **1** and **5** inhibited STAT3 phosphorylation, reduced the level of p50 in the nuclear fraction, and reduced the expression of the tested target genes.

To identify potential points of synthetic chalcones in HCC cells, we examined the effect of thio-derivatives on the level of IKKα/β—an integral point of the NF-κB signaling pathway. Activation of the canonical NF-κB pathway also occurs with the participation of a kappa B kinase inhibitor. However, the precise role of IKKα/β remains unclear in canonical NF-ĸB signaling [35,36]. As observed, in HepG2 cells treated with derivative **1** at 5 µM and 15 μM, the amount of IKKα/β protein in the cytosol was reduced. IKKβ is responsible for the release of NF-κB dimers from the connection with the inhibitor, so the reduction in the level of this protein as a result of the action of thio-derivative **1** may indicate its ability to inhibit the activity of the NF-κB factor.

These results confirm that regulation of the STAT3 and NF-κB signaling pathways may be a promising therapeutic target for HCC using synthetic thio-derivatives of chalcones. The tested compounds modulated the STAT3 and NF-κB pathway in the desired way, i.e., by inhibiting the activity of main transcription factors, leading to changes in the expression of the genes it controls. It suggests that the observed effect of inhibiting the activity of STAT3 and NF-κB factors has consequences at further stages of the signaling pathway. Thio-derivatives of chalcones, including derivative **5** in the DLD-1 cells, decreased the activation of NF-κB and the expression of COX-2. Thus, considering previous studies using colon cancer cells and the current results relating to HCC cells, we can suggest the wide use of synthetic thio-chalcones in the therapy of various types of cancer [11]. Additionally, natural chalcone, xanthohumol, led to the induction of apoptosis in HepG2 cells, and in combination with phenethyl isothiocyanate, downregulated NF-κB activation, reducing binding of its active subunits to DNA, resulting in diminished COX-2 expression [19].

A characteristic feature of cancer cells is the inhibition of apoptosis and deregulation of inflammation-related signaling pathways. The transcription factors NF-κB and STAT3 and the genes under their control play a vital role in these processes. Chronic inflammation resulting from their activation promotes proliferation and creates a characteristic tumor microenvironment that influences the initiation and progression of cancer [33]. Our research allowed us to select from the four thio-derivatives of chalcones tested, compounds **1** and **5**, which showed low toxicity towards non-cancer cells and initiated the apoptotic process in HCC cells.

These compounds in cancer cells also increased the level of the p53 tumor suppressor protein. The observed effects were dose-dependent, but thio-derivative **1** showed activity in HepG2 cells already at a dose of 5 µM. Moreover, thio-chalcones **1** and **5** at higher concentrations (15 µM) influenced both tested pathways—STAT3 and NF-κB. They inhibited the translocation of the transcription factor to the nucleus and influenced the expression of target genes (such as *COX-2*, *Bcl-xL*, and *Bax*) at the transcript and protein levels. Cross-regulation with apoptotic pathways has been postulated because of the vital role of NF-κB in the induction of antiapoptotic genes [29]. Therefore, the NF-κB and STAT3 pathways have already been used as targets in studies seeking new agents to prevent or treat liver cancer [37].

This conception is part of a trend called anakoinosis, and it is an interesting alternative to conventional therapy methods that rely on a single point of attachment or focus on a specific area of the tumor. It has been shown that treatment protocols based on anakoinosis are characterized by low toxicity and are less likely to lead to drug resistance, which distinguishes them from standard therapy methods [12,38]. In the context of HCC, thio-chalcones may modulate STAT3 signaling in several ways. We have proven, among other things, that the tested derivatives can directly block the phosphorylation and translocation of STAT3 to the cell nucleus and, consequently, inhibit the expression of antiapoptotic and proinflammatory proteins. Additionally, chalcones may reduce the production of proinflammatory factors (TNF-α), which weakens STAT3 activation and may further interrupt the feedback loop.

In colon cancer, NF-κB is often excessively activated by pro-inflammatory cytokines via the IkB kinase (IKK) complex, which promotes tumor progression [31]. Inhibition of NF-κB by chalcones can significantly alleviate inflammation and inhibit cancer proliferation. In HCC, the NF-κB pathway may also be overactive, but in this type of cancer, NF-κB has been observed to respond to oxidative stress by regulating the gene expression of reactive oxygen species scavenging proteins.In the case of HCC, chalcones that inhibit NF-κB can reduce oxidative stress and improve the immune response, which leads to different therapeutic effects compared to colorectal cancer [1].

Disorganization of the cell cycle is observed in cancer cells, and the apoptosis process itself is often blocked by the impairment of various signaling pathways. In colorectal cancer, the apoptosis process is stopped by increasing the expression of anti-apoptotic proteins Bcl-2 and Bcl-xL due to excessive activation of NF-κB [39]. This is one of the main mechanisms of resistance to apoptosis. In HCC, STAT3 is strongly associated with the process of apoptosis inhibition, which increases the expression of anti-apoptotic proteins such as Bcl-2 and survivin [40]. Moreover, in HCC, persistent inflammation, which can activate anti- and proapoptotic pathways, usually leads to TNF-α targeting NF-κB activation and promotes cell survival by promoting cytoprotective gene transcription [41].

In summary, the conducted studies have proven that, in addition to the activity of thio-chalcones on colon cancer cells, they also have an effect on various stages of the STAT3 and NF-κB signaling pathways in HCC, depending on specific biological conditions such as inflammation, tumor microenvironment, and apoptosis mechanisms. The obtained results are the basis for using thio-chalones in in vivo studies as potential compounds with anticancer properties. However, further research is necessary to confirm and supplement knowledge regarding the proapoptotic and anti-inflammatory properties of synthetic thio-chalcone derivatives, in particular chalcones **1** and **5**, to use them in the future for prevention and/or treated as substances supporting conventional therapy of liver cancer.

## 4. Materials and Methods

### 4.1. Cell Culture and Viability Assay

The immortalized hepatocytes line THLE-2 (ATCC CRL-2706), and HCC-derived HepG2 cells (ATCC HB 8065) were provided by the American Type Culture Collection (ATCC, Gaithersburg, MD, USA). HepG2 cells were maintained in Dulbecco’s Modified Eagle’s Medium (DMEM, Sigma-Aldrich, St. Louis, MO, USA) containing 10% fetal bovine serum (EURx, Gdansk, Poland) and 1% antibiotic solution (Sigma-Aldrich, St. Louis, MO, USA), while THLE-2 was cultured in BEGM supplemented with BulletKit (Lonza, Basel, Switzerland) and 10% fetal bovine serum, 5 ng/mL EGF, and 70 ng/mL phosphoethanolamine at 37 °C, in a humidified 5% CO_2_ atmosphere.

General procedures for synthesizing novel chalcone thio-derivatives were previously published [11].

To assess the effect of thio-chalcone derivatives on measured parameters, 1 × 10^6^ cells were seeded per 100 mm culture dish, and after 24 h of initial incubation, the cells were treated with 5 and 15 µM 1, 2, 4, and 5 derivatives, 0.1% dimethyl sulfoxide (DMSO) control solution, and with 5 µM BAY 11-7082 (Sigma-Aldrich, St. Louis, MO, USA) inhibitor. Incubation lasted for 24 h, and the cells were harvested.

The effect of the thio-chalcone derivatives on cell viability was assessed using the MTT assay according to a standard protocol. THLE-2 and HepG2 cells were seeded (1 × 10^4^ per well) in 96-well plates. After 24 h of incubation in a complete medium, compounds at various concentrations were added to the cells and further incubated for another 24 h. Cells were then washed twice with phosphate-buffered saline (PBS) and incubated again for 4 h in a medium supplemented with 0.5 mg/mL3-(4,5-dimethylthiazol-2-yl)-2,5-diphenyl-2H-bromide tetrazolium (MTT). The formed formazan crystals were dissolved in acidic isopropanol. The absorbance was measured at 570 and 690 nm. The experiment was repeated three times.

### 4.2. Cell Cycle Distribution

The analysis of the cell cycle distribution was performed using the Muse Cell Cycle Kit (Merck, Darmstadt, Germany) according to the manufacturer’s protocol. Briefly, cells (3 × 10^5^ per well) were seeded and grown on 6-well plates for 24 h before stimulation. Then, the tested compounds were added, and cells were incubated for 24 h. Topotecan (1500 nM)-treated cells were used as a positive control for cell cycle arrest. Subsequently, cells were harvested by trypsinization, washed with PBS buffer, fixed in ice-cold 70% ethanol, and frozen until staining at −20 °C. After overnight storage, cells were washed with cold PBS buffer, stained with propidium iodide in the presence of RNAase A, and, after 30 min of incubation at room temperature and in the dark, the fluorescence signal was analyzed by flow cytometry using the Muse^®^ Cell Analyzer and Muse^®^ 1.5 Analytical Software (Merck, Darmstadt, Germany).

### 4.3. Apoptosis Analysis

The Muse^®^ Annexin V & Dead Cell Kit (Merck, Darmstadt, Germany), containing one of the known apoptosis markers, phosphatidylserine externalization, was used to assess the apoptosis profile of HepG2 line cells treated with thio-chalcones. 7-aminoactinomycin D (7-AAD) staining also differentiated early and late apoptotic cells. Cells (3 × 10^5^ per well) were seeded in 6-well plates, which were first incubated for 24 h and then cultured for another 24 h in the presence of the test compounds and Topotecan (1500 nM), which was a positive control for apoptosis induction. Cells were then harvested by trypsinization, resuspended in fresh medium, and stained with Annexin V and 7-AAD solution. The whole thing was incubated for 20 min in the dark at room temperature. The analysis was performed by flow cytometry using the Muse^®^ Cell Analyzer and Muse^®^ 1.5 Analytical Software (Merck, Darmstadt, Germany).

### 4.4. Cytosolic and Nuclear Fractions Preparation

According to the manufacturer’s protocol, the subcellular extracts from HepG2 cells were prepared using the Nuclear/Cytosol Fractionation Kit (BioVision Research, Milpitas, CA, USA). Cells were centrifuged at 600× *g* for 5 min at 4 °C, and samples were kept on ice to preserve cytosolic enzyme activity. After discarding the supernatant, extraction buffer (CEB-A) with dithiothreitol (DTT) and protease inhibitors was added. The mixture was shaken, incubated on ice, and then the extraction buffer (CEB-B) was added for the cytoplasmic fraction, which was stored at −80 °C. Extraction buffer (NEB) with DTT and protease inhibitors was added to the remaining sediment, incubated, and centrifuged to isolate the nuclear fraction. The nuclear fraction was also stored at −80 °C.

### 4.5. Bead-Based Immunoassay on the Luminex MAGPIX Instrument

A magnetic bead-based immunoassay was performed on the Luminex-MAGPIX multiplex immunoassay system. Data were analyzed with MILLIPLEX^®^ Analyst 5.1 software (EMD Millipore, Burlington, MA, USA). The panel that we performed quantitated the following proteins in the cytosolic fractions of HepG2 cells: CREB, phospho-CREB, JNK, phospho-JNK, ERK, phospho-ERK, Akt, phospho-Akt, p38, phospho-p38, p70S6K, and phospho-p70S6K according to the manufacturer’s instructions. The magnetic bead panel, with high-sensitivity antibodies, was obtained from Merck (Darmstadt, Germany). Using Luminex xMAP technology with different fluorescent colors, a multiplex test based on microspheres was detected on a compatible MAGPIX camera. Cytosolic fractions were suspended in MILLIPLEX^®^ MAP buffer. The bead suspension was added to each well of a 96-well plate, and then samples were added to the wells and incubated overnight at 2–8 °C on a shaker protected from light. The plate was washed with 2× buffer, and then 1X MILLIPLEX^®^ MAP detection antibodies were added. After shaking for 1 h at room temperature, the antibodies were removed, and 1X MILLIPLEX^®^ MAP Streptavidin/Phycoerythrin (SAPE) was added. Then, the MILLIPLEX^®^ MAP Amplification Buffer was added to each well and shaken for 15 min. The beads were suspended in MILLIPLEX^®^ MAP buffer, and each microsphere was identified with a MAGPIX Luminex Analyzer (Merck, Darmstadt, Germany); the results were calculated from the reporters’ fluorescent signals. Mean fluorescence intensities were quantified with the xPonent 4.2 software (Luminex Corporation, Austin, TX, USA). The raw MFI results for the tested protein levels were converted relative to the control of DMSO-treated cells.

### 4.6. Western Blot Analysis

Cytosolic extracts (for β-actin, NF-κBp65, NF-κBp50, COX-2, iNOS, STAT3, Bax, Bcl-xl, IKKα/β, p53, and TNF-α) or nuclear extracts (for lamin, NF-κBp65, NF-κBp50, STAT3, and p-STAT3) were separated on 7.5%, 10%, or 12% SDS–PAGE slab gels. Then, the proteins were transferred to a nitrocellulose Immobilon-P membrane. After blocking for 2 h with 10% skimmed milk, proteins were incubated with primary antibodies (Abcam, Cambridge, UK and Santa Cruz Biotechnology, Dallas, TX, USA) against monoclonal mouse anty-p53 (ab1101), mouse anty-Bcl-xL (ab77571), mouse anti-TNF-α (sc-130349) and polyclonal rabbit anti-βactin (sc-sc-7210) rabbit anti-lamin (sc-206800), rabbit anti-NF-κB p65 (sc-7151), rabbit anti-NF-κB p50 (sc-114), rabbit anty-IKKα/β (sc-7607), rabbit anti-COX-2 (sc-376861), rabbit anty-iNOS (sc–651), rabbit anty-STAT3 (sc–483), goat anty-p-STAT3 (sc–7993), and mouse anti-Bax (sc-493). Alkaline phosphatase (AP)-labeled anti-rabbit IgG, anti-goat IgG, and anti-mouse IgG secondary antibodies (Santa Cruz Biotechnology, Dallas, TX, USA), as well as horseradish peroxidase (HRP)-conjugated anti-mouse IgG (Boster Bio, Pleasanton, CA, USA) secondary antibodies were used in the staining reaction. Bands were visualized by the AP Conjugate Substrate Kit NBT/BCIP or the chemiluminescent HRP substrate of the Clarity ECL Kit (Bio–Rad Laboratories, Hercules, CA, USA). The amount of immunoreactive product in each lane was determined using a ChemiDoc Imaging System (BioRad Laboratories, Hercules, CA, USA).

### 4.7. Preparation of RNA and cDNA Synthesis

Total RNA from the cells was extracted using a Universal RNA Purification Kit (EURx, Gdansk, Poland) according to the manufacturer’s instructions. Cells were centrifuged at 1000× *g* for 5 min, and 400 µL of RL lysis buffer with β-mercaptoethanol was added to the sediment. The lysate was processed through a mini-column, followed by ethanol and washing buffers. RNA was eluted with RNase-free water. RNA was then reverse transcribed into cDNA using the RevertAid First Strand cDNA Synthesis Kit. The RNA was incubated with primers at 65 °C, followed by the addition of reverse transcriptase and other reagents. cDNA synthesis occurred at 42 °C for 60 min, and samples were stored at −80 °C.

### 4.8. Quantitative Real-Time PCR (qRT–PCR)

Total RNA from cells was isolated with the GeneMatrix Universal DNA/RNA/Protein Purification Kit (EurX, Gdansk, Poland). For the qRT-PCR analyses, the Maxima SYBR Green I/ROX qPCR Master Mix (2×) (Thermo Fisher Scientific, Waltham, MA, USA) and the thermal cycler LightCycler (Roche, Penzberg, Germany) were used. The protocol started with 5 min of enzyme activation at 95 °C, followed by 40–50 cycles at 95 °C for 15 s, 56 °C for 20 s, 72 °C for 40 s, and final elongation at 72 °C for 5 min. Melting curve analysis was used for amplicon verification. TATA-box-binding protein (TBP) and porphobilinogen deaminase (PBGD) expression levels were used to normalize the data. The Pfaffl comparative method was used to calculate fold change. Primers were designed using Beacon Designer software (the sequences of primers used to analyze genes are listed in Table 3).

### 4.9. Statistical Analysis

Statistical analysis was performed using GraphPad InStat version 3.10 (GraphPad Software, San Diego, CA, USA), assuming the significance level of changes to be *p* < 0.05. Student’s *t*-test was used to assess the similarity of the experimental groups to the control. Additionally, Dunnett’s post hoc test was used to assess the significance of the differences between experimental groups and the group treated with BAY 11-7082 (Sigma-Aldrich, St. Louis, MO, USA).

## 5. Conclusions

In summary, we identified two compounds, **1** and **5**, as the most potent synthetic thio-chalcones for further study. Their anticancer effect appears to be associated with inhibiting proinflammatory and proapoptotic kinases CREB, Akt, p38, signaling mediated by NF-κB, STAT3, and proteins such as p53, TNF-α, Bax, and Bcl-xL. These findings provide a new biochemical basis for applying chalcone thio-derivatives to treat HCC as a part of the anakoinosis conception.

## Figures and Tables

**Table 1:** The chemical structure of the analyzed synthetic thio-chalcone derivatives.

The Name of the Synthetic Chalcone Thio-Derivatives	Symbol	Structural Formula
**3-(4-methoxy-3-methylthiophenyl)-1-(3,4,5-trimethoxyphenyl)-prop-2-en-1-one**	**1**	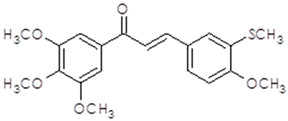
**3-(3-methoxy-4-methylthiophenyl)-1-(3-bromo-4,5-dimethoxyphenyl)-prop-2-en-1-one**	**2**	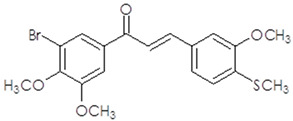
**3-(4-methylthiophene)-1-(3-bromo-4,5-dimethoxyphenyl)-prop-2-en-1-one**	**4**	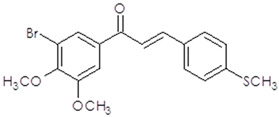
**3-(3-methoxy-4-methylthiophenyl)-1-(3-bromo-5-methoxy-4-methylthiophene)-prop-2-en-1-one**	**5**	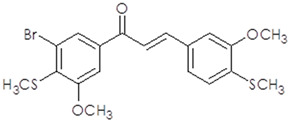

**Table 2:** The IC50 values for a 24 h treatment of THLE-2 and HepG2 cell lines with synthetic thio-chalcone derivatives (**1**, **2**, **4**, and **5**).

Chalcone Thio-Derivative	THLE-2 IC50 Value ± SEM (µM)	HepG2 IC50 Value ± SEM (µM)
**1**	24 ± 0.58	15 ± 1.54
**2**	18 ± 2.2	13 ± 2.28
**4**	17 ± 0.52	15 ± 1.98
**5**	26 ± 1.35	16 ± 0.97

**Table 3:** Sequences of primers used in the qRT-PCR reaction (Laboratory of Sequencing and Synthesis of Oligonucleotides of the Institute of Biochemistry and Biophysics of the Polish Academy of Sciences, Poland).

Gene	Primer F (5′ → 3′)	Primer R (5′ → 3′)
*NF-κB p50*	5′ATCATCCACCTTCATTCTCAA	5′AATCCTCCACCACATCTTCC
*NF-κB p65*	5′CGCCTGTCCTTTCTCATC	5′ACCTCAATGTCCTCTTTCTG
*COX-2*	5′CGCCTGTCCTTTCTCATC	5′CAGCCCGTTGGTGAAAGC
*iNOS*	5′AGGAGATGCTGAACTACG	5′GGATGGTGACTCTGACTC
*STAT3*	5′GCTTCTCCTTCTGGGTCTG	5′AGGCTTAGTGCTCAAGATGG
*Bcl-xL*	5′AAGCGTAGACAAGGAGATGC	5′CAGCGGTTGAAGCGTTCC
*Bax*	5′GCTTCAGGGTTTCATCCAG	5′GGCGGCAATCATCCTCTG
**References Genes**		
*PBGD*	5′TCAGATAGCATACAAGAGACC	5′TGGAATGTTACGAGCAGTG
*TBP*	5′GGCACCACTCCACTGTATC	5′GGGATTATATTCGGCGTTTCG

## Data Availability

The original contributions presented in the study are included in the article/Supplementary Materials, further inquiries can be directed to the corresponding author/s.

## Supplementary Materials

The following supporting information can be downloaded at: https://www.mdpi.com/article/10.3390/ijms251910739/s1.
