# Supplementary material for: Modulatory Effects of Chalcone Thio-Derivatives on NF-κB and STAT3 Signaling Pathways in Hepatocellular Carcinoma Cells: A Study on Selected Active Compounds
Source: Int J Mol Sci. 2024 Oct 5;25(19):10739. doi: 10.3390/ijms251910739 (PMC11476945; doi:10.3390/ijms251910739)
Supplement: Supplementary file 1 [file ijms-25-10739-s001.zip › ijms-3189764-supplementary.pdf]

Supplementary Figures

Article

Modulatory Effects of Chalcone Thio-Derivatives on NF-κB and STAT3 Signaling Pathways in Hepatocellular Carcinoma Cells: A Study on Selected Active Compounds

Katarzyna Papierska[1*] , Eliza Judasz[1], Wiktoria Tonińska[1], Maciej Kubicki[2], Violetta Krajka-Kuźniak[1]

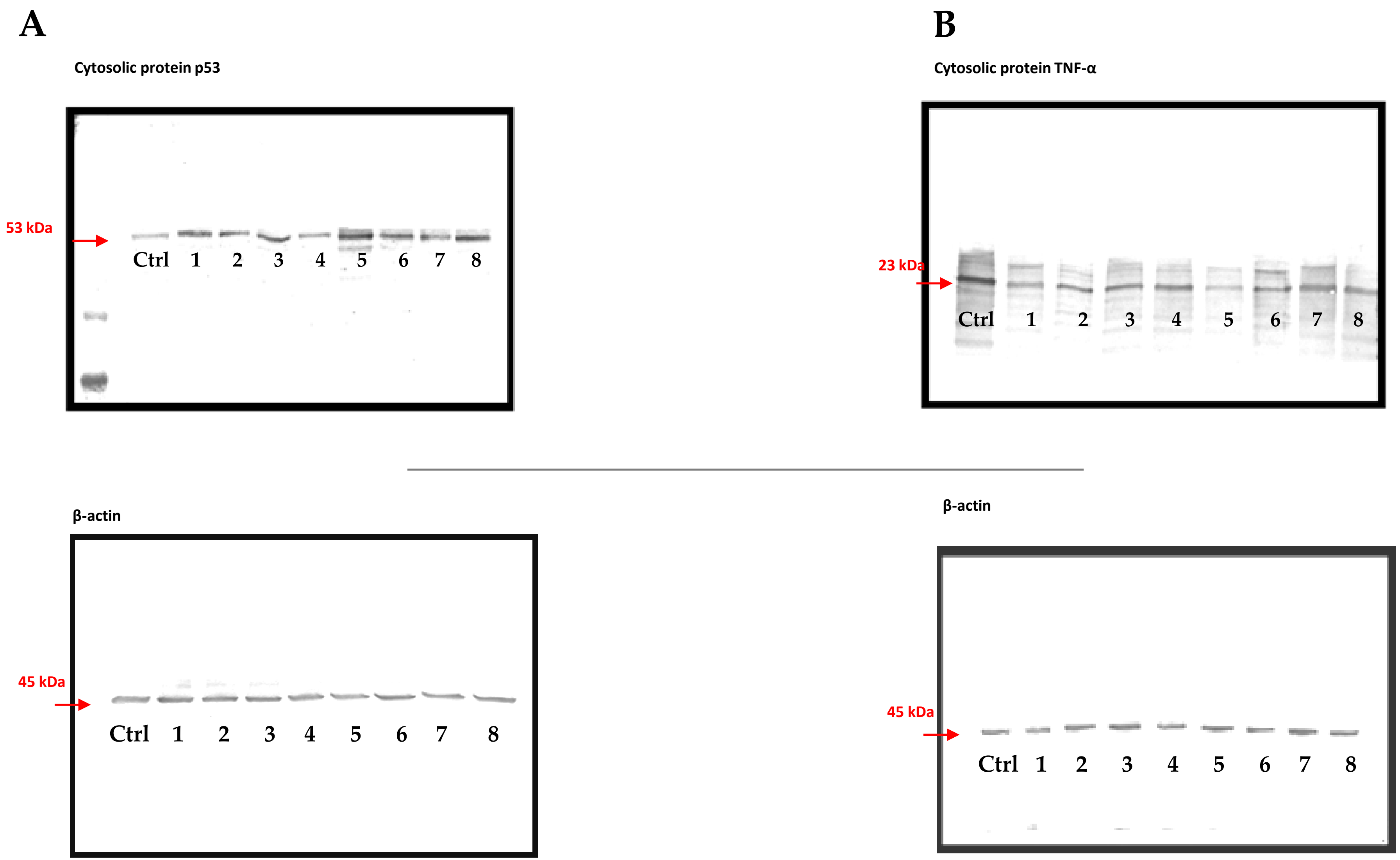

Figure S1. The representative immunoblots respectively for Figure 4. ***Effect of the tested synthetic thio-chalcone derivatives (1, 2, 4, and 5) on the protein level of p53 (panel A) and TNF-α (panel B) in the HepG2 cell line.*** Data were normalized against the level of β-actin. **Ctrl –** Control, **1** – 1/5, **2** – 2/5, **3**- 4/5, **4** – 5/5, **5** – 1/15, **6** – 2/15, **7** – 4/15, **8** – 5/15.

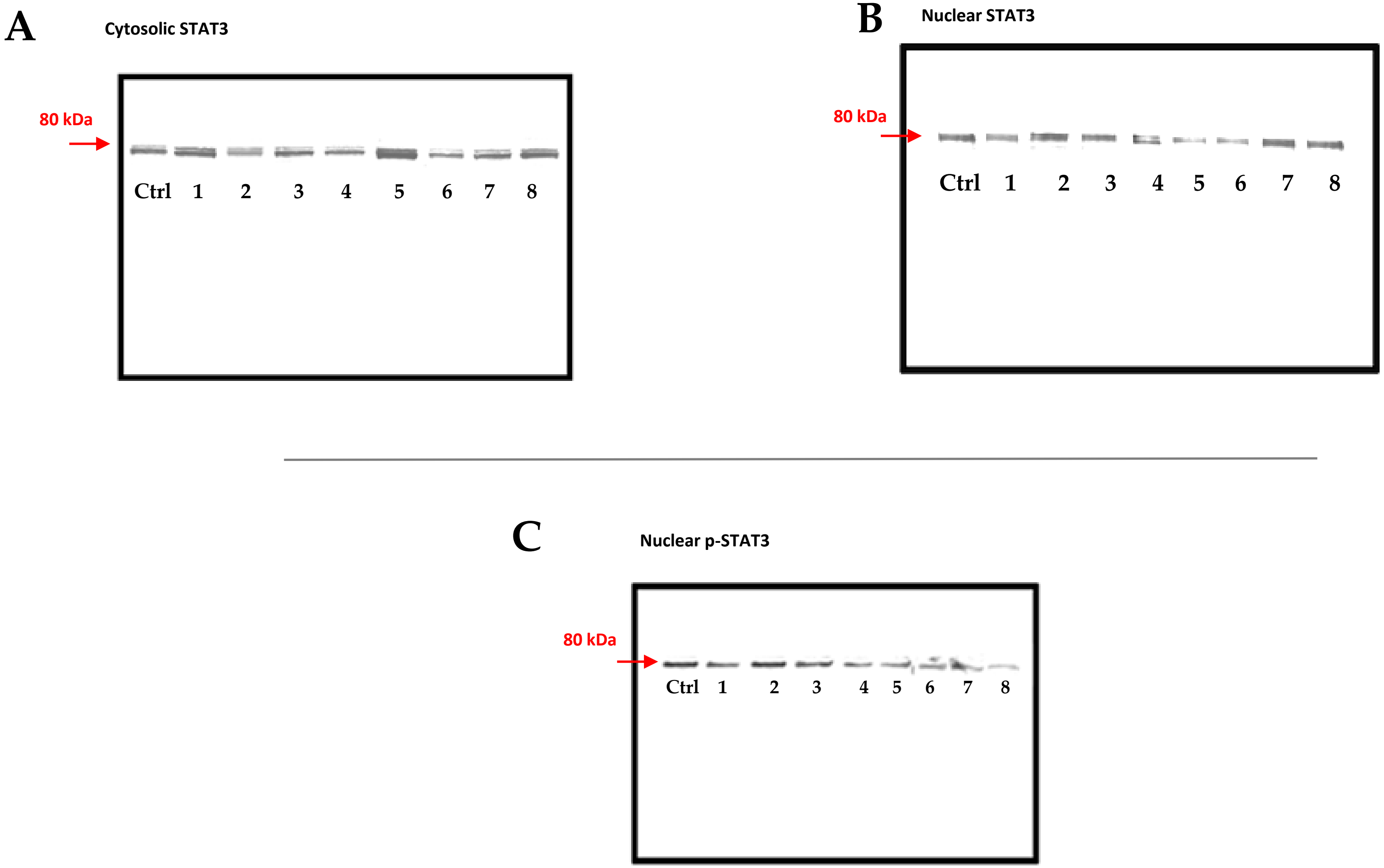

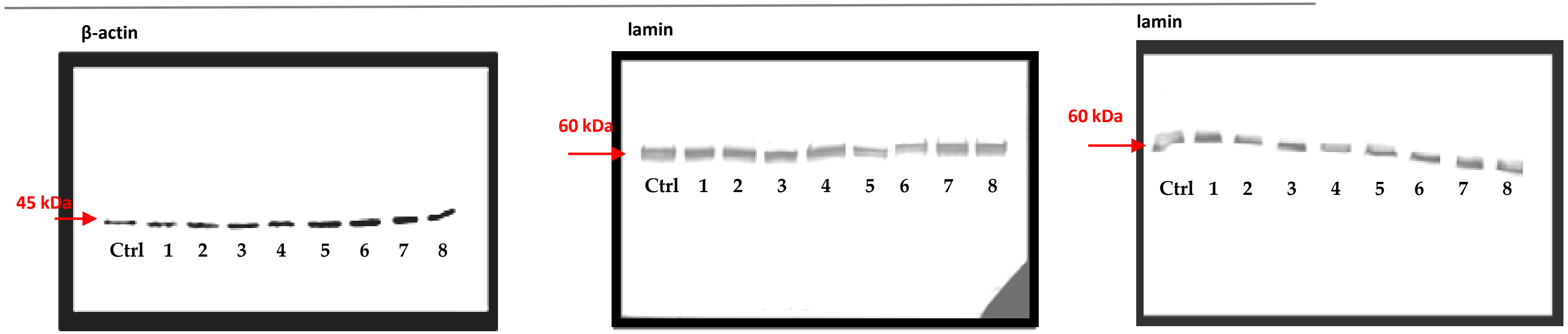

Figure S2. The representative immunoblots respectively for Figure 6. *Effect of the tested synthetic thio-chalcone derivatives (1, 2, 4, and 5) on the protein level of STAT3 in cytosolic fraction (panel A), in nuclear fraction (panel B) and phospho-STAT3 (panel C) in the HepG2 cell line.* Data were normalized against the level of β-actin and lamin. **Ctrl** – Control, **1** – 1/5, **2** – 2/5, **3**- 4/5, **4** – 5/5, **5** – 1/15, **6** – 2/15, **7** – 4/15, **8** – 5/15.

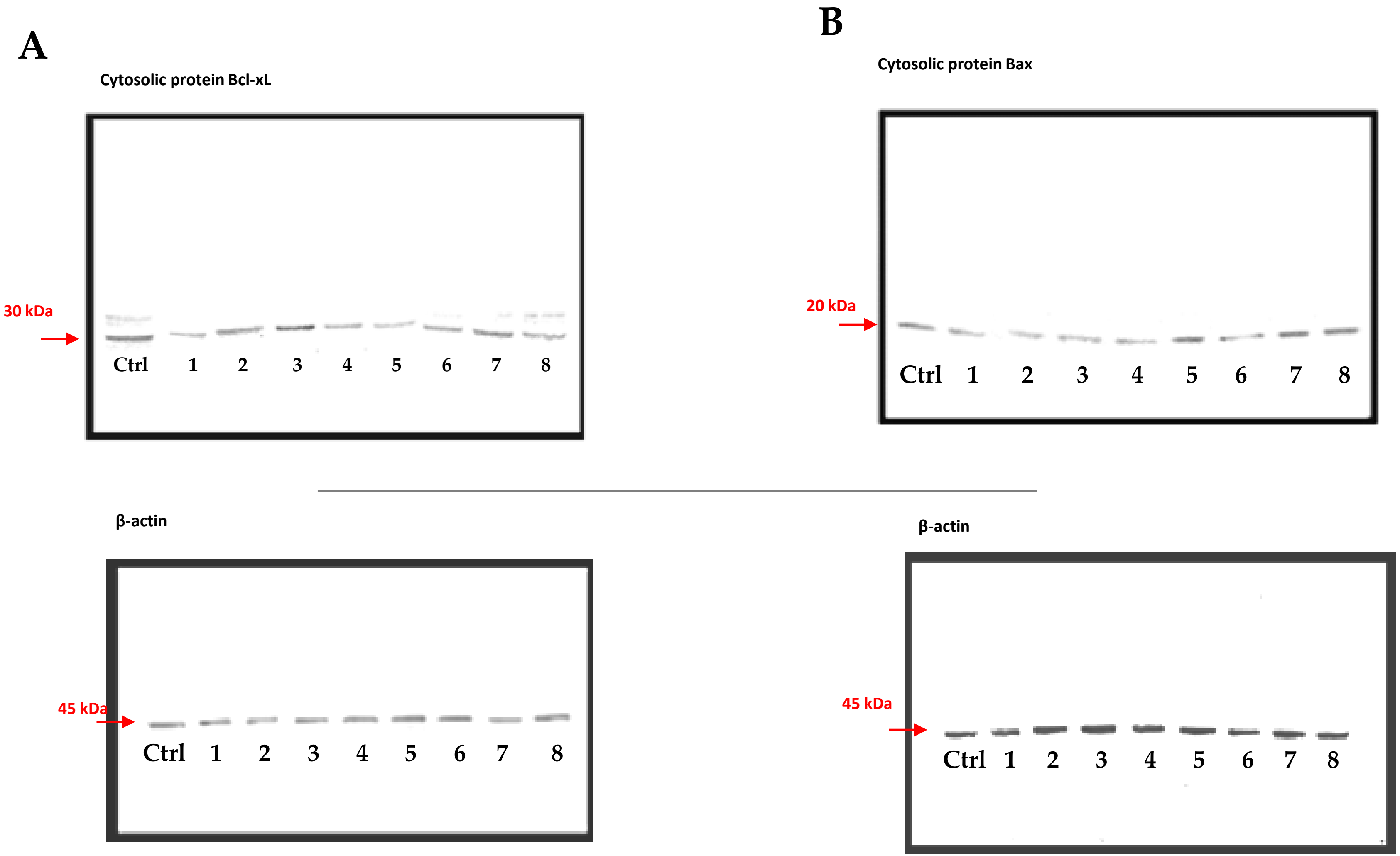

Figure S3. The representative immunoblots respectively for Figure 7. ***Effect of the tested synthetic thio-chalcone derivatives (1, 2, 4, and 5) on the expression of STAT3 and selected target genes in the HepG2 cell line.*** Bcl-xL protein level (panel A) and Bax protein level (panel B). Data were normalized against the level of β-actin. **Ctrl –** Control, **1** – 1/5, **2** – 2/5, **3**-4/5, **4** – 5/5, **5** – 1/15, **6** – 2/15, **7** – 4/15, **8** – 5/15.

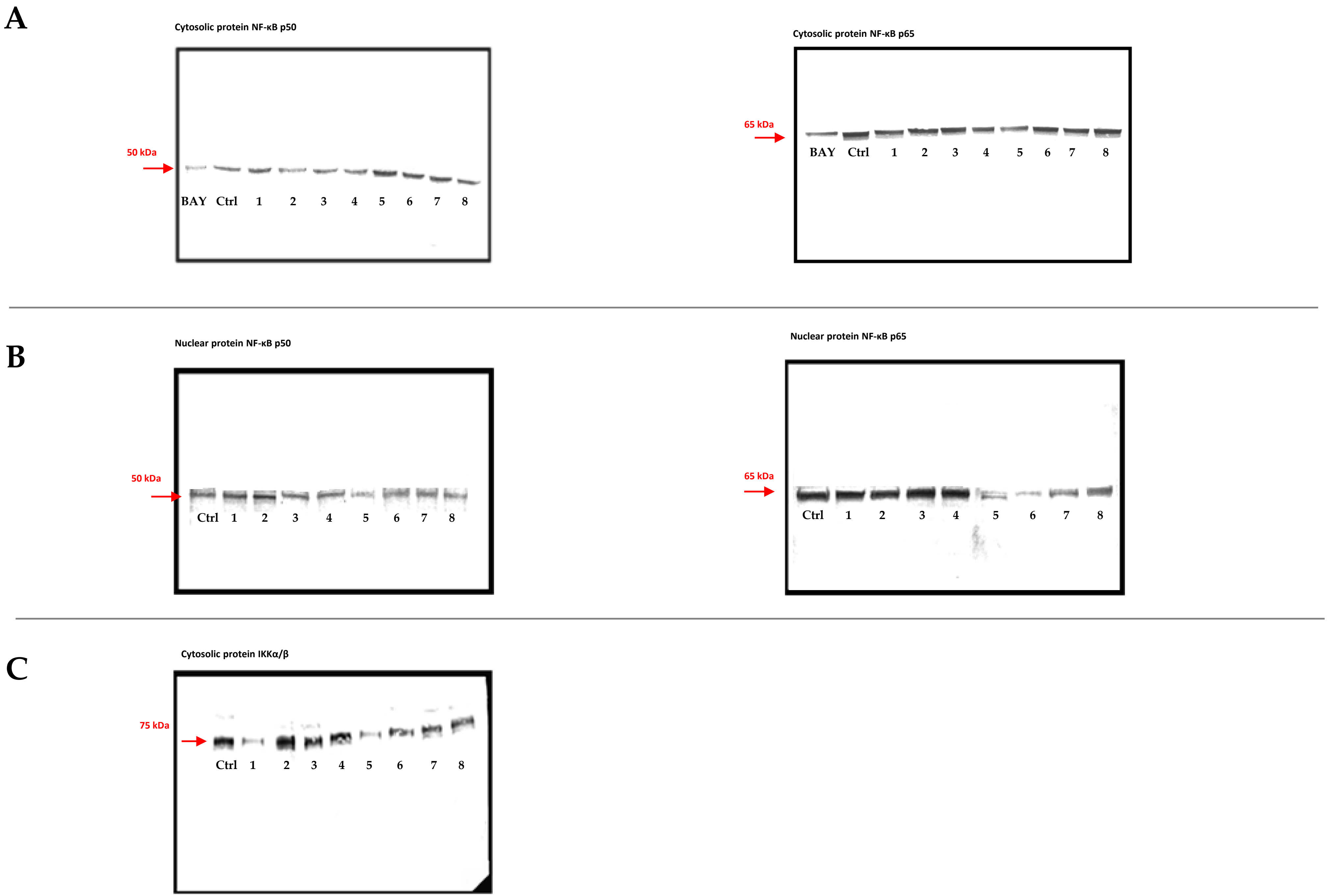
A
Cytosolic protein NF-κB p50
50 kDa
BAY Ctrl 1 2 3 4 5 6 7 8
Cytosolic protein NF-κB p65
65 kDa
BAY Ctrl 1 2 3 4 5 6 7 8
B
Nuclear protein NF-κB p50
50 kDa
Ctrl 1 2 3 4 5 6 7 8
Nuclear protein NF-κB p65
65 kDa
Ctrl 1 2 3 4 5 6 7 8
C
Cytosolic protein IKKα/β
75 kDa
Ctrl 1 2 3 4 5 6 7 8

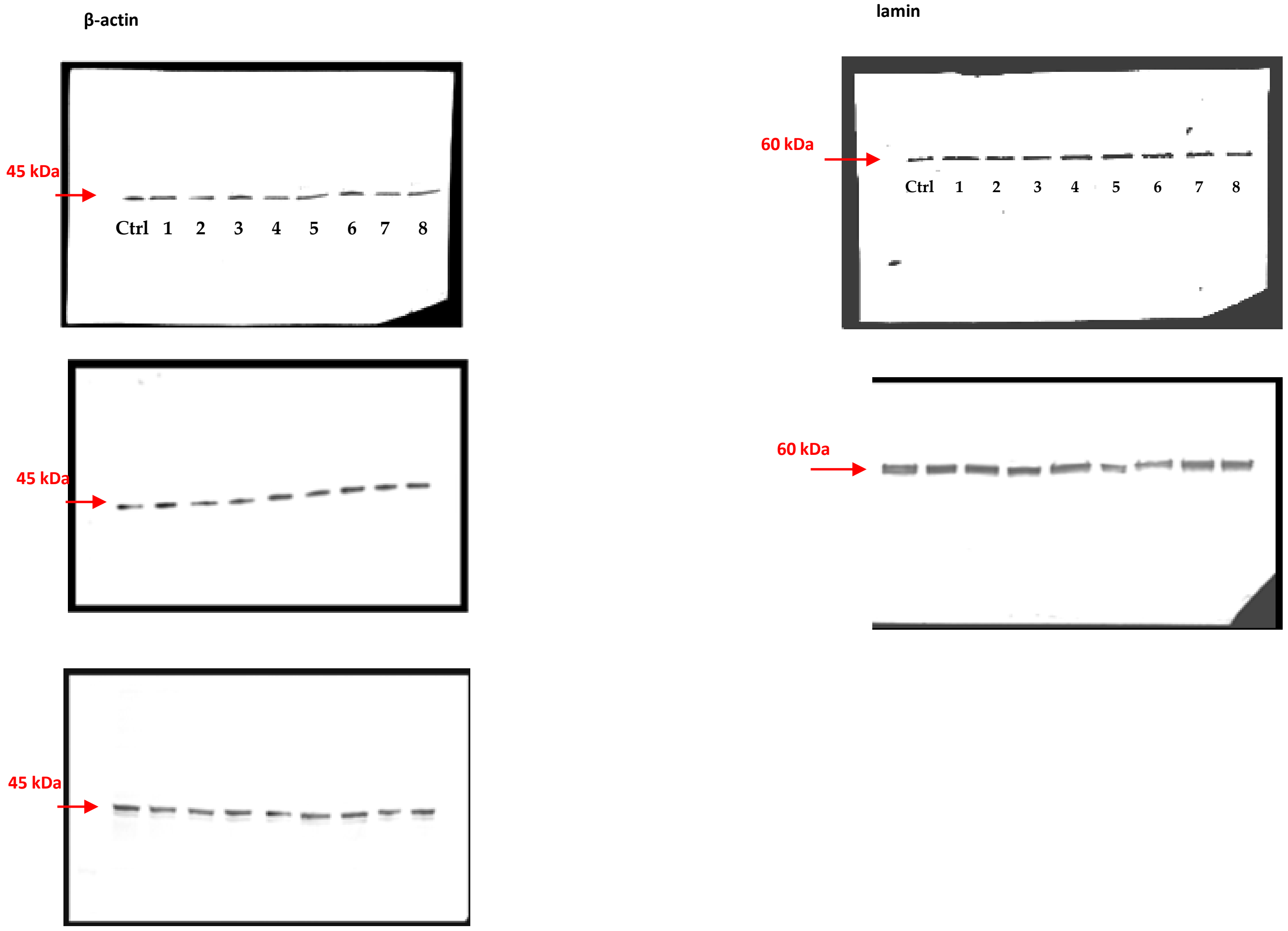

Figure S4. The representative immunoblots respectively for Figure 8. *Effect of the tested synthetic thio-chalcone derivatives (1, 2, 4, and 5) on the protein level of NF- κB p50 and p65 subunits in cytosolic fraction (panel A), in nuclear fraction (panel B) and IKKα/β in cytosolic fraction (panel C) in the HepG2 cell line.* Data were normalized against the level of β-actin and lamin. BAY - BAY/5, **Ctrl –** Control, **1** – 1/5, **2** – 2/5, **3**- 4/5, **4** – 5/5, **5** – 1/15, **6** – 2/15, **7** – 4/15, **8** – 5/15.

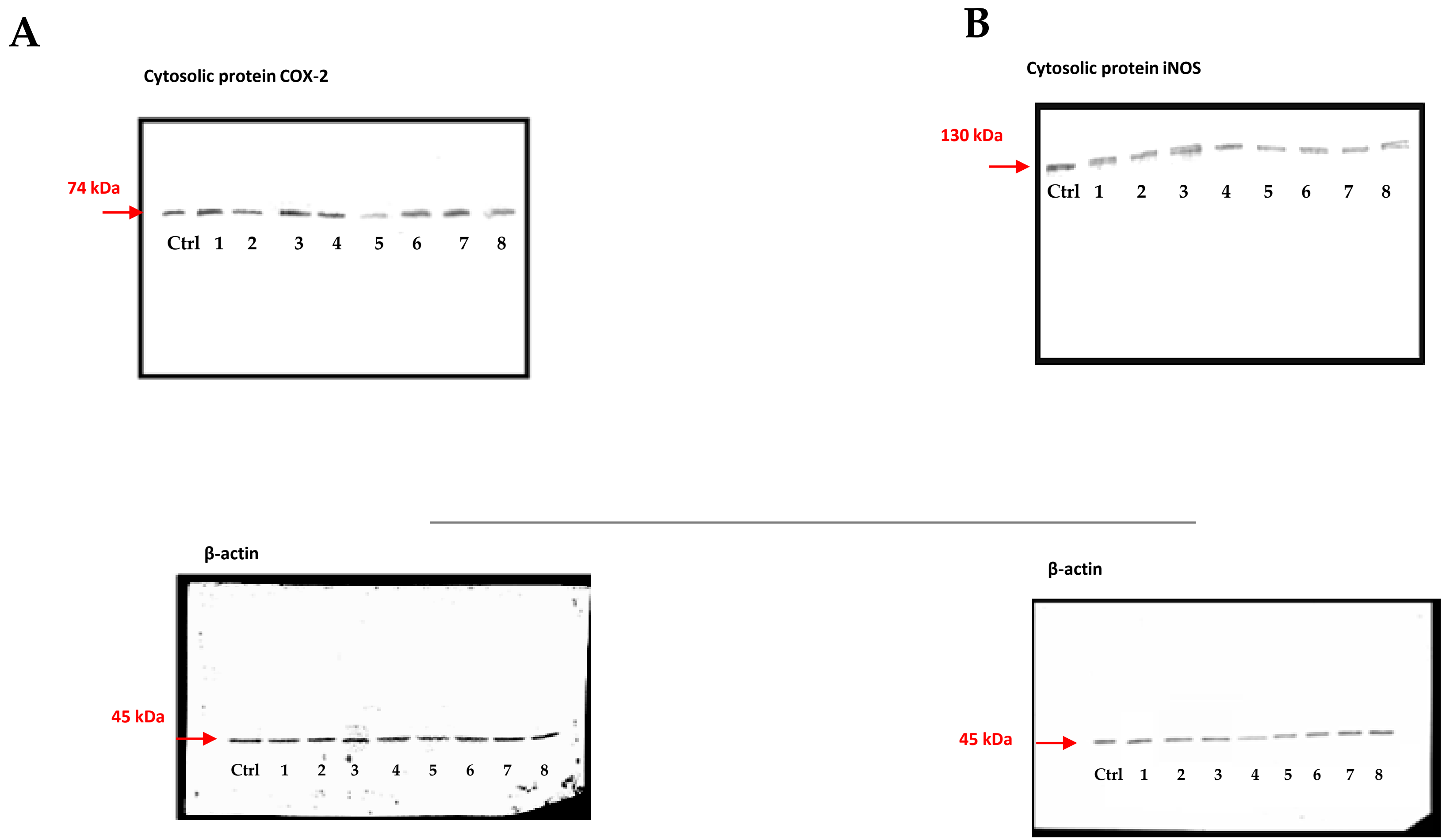

Figure S5. The representative immunoblots respectively for Figure 9. ***Effect of the tested synthetic thio-chalcone derivatives (1, 2, 4, and 5) on the expression of NF-κB and selected target genes in the HepG2 cell line.*** COX-2 protein level in panel (A) and iNOS protein level in panel (B). Data were normalized against the level of β-actin. **Ctrl –** Control, **1** – 1/5, **2** – 2/5, **3**- 4/5, **4** – 5/5, **5** – 1/15, **6** – 2/15, **7** – 4/15, **8** – 5/15.
